# Hemopoietic and Lymphoid Progenitor Cells in Human Umbilical Cord Blood

**DOI:** 10.1155/1994/83780

**Published:** 1994

**Authors:** F. M. Cicuttini, A. W. Boyd

**Affiliations:** Lions Clinical Cancer Research Laboratory Walter and Eliza Hall Institute of Medical Research PO Royal Melbourne Hospital Melbourne 3050 Australia

**Keywords:** Human umbilical cord blood, hemopoiesis, T cells, B cells, natural killer cells

## Abstract

Human umbilical cord blood, which in the past was discarded with the placental tissue, provides a convenient source of fetal hemopoietic cells for scientific analysis and clinical use. Cord blood cells are immature compared to analogous populations in adult peripheral blood. Cord blood B lymphocytes display unique phenotypic and functional characteristics. The antigens CD1C, CD38, CD5, and CD23, although normally expressed on only a small percentage of circulating B cells in adults, are highly expressed on cord blood B cells. Recent studies have demonstrated that whereas cord blood B cells are functionally naive, their potential is similar to that of adult B cells if optimal T-cell help is available. Thus, the failure of B-cell responses in cord blood is due to the T cells. The functional abnormalities of T cells from newborns can be summarized as a dominance of the effects of TH0 cells. Thus, the cytokines produced are immunosuppressive rather than mediating helper activity for B cells. NK activity in cord blood is also depressed compared to that in adults. Cord blood is a very rich source of hemopoietic progenitor cells. The spectrum of progenitors shows a predominance of early progenitor cells when compared with bone marrow. These cells provide an alternative source to adult bone marrow for stem cells to use for hemopoietic reconstitution and as targets in the treatment of hereditary deficiencies by gene therapy. These features make cord blood a unique research tool to investigate hemopoietic ontogeny and a unique clinical tool for transplantation.


*Developmental Immunology*, 1994, Vol 4, pp. 1–11
Reprints available directly from the publisher
Photocopying permitted by license only

© 1994 Harwood Academic Publishers GmbH
Printed in Singapore

### Hemopoietic and Lymphoid Progenitor Cells in Human Umbilical Cord Blood

F.M. CICUTTINI* and A.W. BOYD

*Lions Clinical Cancer Research Laboratory, Walter and Eliza Hall, Institute of Medical Research, PO Royal Melbourne Hospital, 3050*

Human umbilical cord blood, which in the past was discarded with the placental tissue, provides a convenient source of fetal hemopoietic cells for scientific analysis and clinical use. Cord blood cells are immature compared to analogous populations in adult peripheral blood. Cord blood B lymphocytes display unique phenotypic and functional characteristics. The antigens CD1C, CD38, CD5, and CD23, although normally expressed on only a small percentage of circulating B cells in adults, are highly expressed on cord blood B cells. Recent studies have demonstrated that whereas cord blood B cells are functionally naive, their potential is similar to that of adult B cells if optimal T-cell help is available. Thus, the failure of B-cell responses in cord blood is due to the T cells. The functional abnormalities of T cells from newborns can be summarized as a dominance of the effects of TH0 cells. Thus, the cytokines produced are immunosuppressive rather than mediating helper activity for B cells. NK activity in cord blood is also depressed compared to that in adults. Cord blood is a very rich source of hemopoietic progenitor cells. The spectrum of progenitors shows a predominance of early progenitor cells when compared with bone marrow. These cells provide an alternative source to adult bone marrow for stem cells to use for hemopoietic reconstitution and as targets in the treatment of hereditary deficiencies by gene therapy. These features make cord blood a unique research tool to investigate hemopoietic ontogeny and a unique clinical tool for transplantation.

KEYWORDS: Human umbilical cord blood, hemopoiesis, T cells, B cells, natural killer cells.

### INTRODUCTION

In early human development, as in that of other mammals, it is believed that hemopoiesis begins in the yolk sac. Primitive progenitors are thought to migrate to fetal liver and finally to the bone marrow, which remains the primary source of hemopoietic cells throughout adult life. In keeping with this model, erythropoietic activity is present in the liver of the 6-week-old embryo with granulopoiesis occurring a 7 weeks (Bloom and Bartelmez, 1940). The fetal liver is the primary source of red cells from the 9th to the 24th week of gestation, whereas eythropoiesis and granulopoiesisis are first evident in the bone marrow of the 10- to 11-week-old embryo (Bloom and Bartelmez, 1940). Hemopoietic activity increases rapidly in the ensuing weeks with the marrow becoming the major site of hemopoiesis after the 24th week of gestation. Although lymphopoiesis has not been observed in the yolk sac, it is present in the lymphoid plexuses at 9 weeks and in the lymph glands by 11 weeks of gestation (Gupta et al., 1976).

Although transfer of hemopoietic activity from the liver to the bone marrow is virtually complete at the time of birth, it has been shown that human umbilical cord blood (HUCB) is rich in hemopoietic progenitor cells (Broxmeyer et al., 1989). Human umbilical cord blood, which in the past was discarded with the placental tissue, provides a convenient source of fetal hemopoietic cells for scientific analysis and clinical use. In this review, we will examine the properties of lymphohemopoietic cells in HUCB and outline the differences between these cells and those found in adult peripheral blood (Table 1). We will consider the hemopoietic progenitor cells, B, T lymphocytes, and NK cells in detail.

*Corresponding author.

TABLE 1
A Comparison of Cell Populations in Cord Blood versus Adult Blood

| | Cord blood | Adult blood | References |
|---|---|---|---|
| Haemopoietic progenitor cells | | | |
| % of total mononuclear cells expressing CD34 | 1 | <0.1 | Cicuttini et al., 1992 |
| B cells | | | |
| Pre-B cells (% of total lymphocytes) | 0.7 | 0.2 | Okino, 1987 |
| B cells (% of total lymphocytes) | 11.4 | 5.4 | Okino, 1987 |
| % B cells expressing: | | | |
| CD1c | 90 | 15–20 | Small et al., 1989 |
| CD23 | 70 | 10 | Small et al., 1989 |
| CD38 | 90 | 15–20 | Small et al., 1989 |
| T cells | | | |
| % CD4+ T cells coexpressing: | | | |
| CD29 | <10 | 50 | Clement et al., 1990 |
| CD38 | >90 | 10–20 | Clement et al., 1990 |
| CD45RA. | >90 | 40–50 | Clements ėt al., 1990 |
| NK cells | | | |
| % expressing: | | | |
| CD16 | 80–90 | 80–90 | Tarakkanan and Saksela, 1982 |
| CD57 | <1 | 50–60 | Abo et al., 1982 |

### Human Umbilical Cord Blood as a Source of Hemopoietic Progenitor Cells

Circulating blood cells are derived and replaced by the hemopoietic stem and progenitor cell pools (Williams et al., 1987). Hemopoietic progenitor cells are present in human adult bone marrow but also in adult peripheral blood and umbilical cord blood (Caracciola et al., 1989). All human hemopoietic progenitor cells as well as bone marrow reconstituting (stem) cells express CD34-antigens (Civin et al., 1987; Berensson et al.; 1988a, 1988b).

As the primary site of production of stem/progenitor cells in human adults is the bone marrow (Allen and Dexter, 1984), autologous or major histocompatibility complex-matched bone marrow transplantation has become the standard means of clinical hemopoietic reconstitution. However, HUCB has been used to effect hematological reconstitution, with sufficient stem cells available in the cord blood obtainable from a single placenta to reconstitute adult (Broxmeyer et al., 1989). In this study, three unseparated cord blood samples were shown to contain $1.9 \pm 1.5 \times 10^6$ granulocyte-macrophage colony-forming cells (CFU-GM), which compares favorably with the minimum CFU-GM associated with successful engraftment in adults receiving marrow autografts (mean [range] 1.3 [0.3–6.3] $\times 10^6$) Douay et al., 1986) and allografts (2.6 [0.5–5.5] $\times 10^6$) (Ma et al., 1987). Although normal adult blood has had some use as an alternative source (To and Juttner, 1987), in practice, the content of stem/progenitor cells is so low that multiple leukophoreses are necessary (To and Juttner, 1987). This is also a limitation in using this source for *in vitro* studies, although the number of progenitor cells has been reported to increase transiently in patients undergoing intense chemotherapy or following G-CSF infusion (Juttner et al., 1990).

In general, numbers of hemopoietic progenitor cells are monitored by clonogenic assays. These allow for the growth and differentiation of progenitor cells, resulting in hemopoietic colonies after various periods of culture (Lajtha, 1979). *In vitro* culture of HUCB has demonstrated multipotential (CFU-GEMM), erythroid (BFU-E), and granulocyte-macrophage (CFU-GM) progenitor cells (Leary et al., 1984). A proportion of colonies also contains progenitors that form secondary colonies when replated in a secondary agar culture. This property suggests that the colony arises from a single cell with limited self-renewal properties. The frequency of cord blood progenitors (number of colonies formed/number of cells plated) equals or exceeds that of marrow and greatly surpasses that of adult blood. Progenitor cells from HUCB can be maintained for several weeks in long-term liquid culture systems, suggesting their production from more primitive cells (Salahuddin et al., 1981; Smith and Broxmeyer, 1986).

We recently described the purification of highly purified CD34+ progenitor cells from HUCB by immunodepletion followed by positive FACS sorting (Cicuttini et al., 1992a). This resulted in > 100-fold enrichment of colony-forming cells (CFC). Cord blood progenitor cells were shown to be skewed to very early cells in that cord blood CD34+ cells grown in the presence of stem-cell factor (SCF), and optimum growth factors resulted in a very high proportion (50–80%) of mixed colonies (CFU-GEMM). This was in agreement with another study examining progenitors from cord blood (Broxmeyer et al., 1991). However, significantly fewer CFU-GEMM were observed when bone marrow was cultured under similar conditions (Anderson et al., 1990; Nocka et al., 1990; McNeice et al., 1991). These findings suggest that the stem/progenitor cell pool in cord blood is weighted toward very early progenitor cells.

In our studies, we also examined the ability of CFU-GEMM colonies to reclone. That is, the ability of cells harvested from the CFU-GEMM colonies, on reculturing in the presence of the same hemopoietic growth factors, to form daughter colonies. This would indicate that they contain more immature CFC than have been described for CFU-GEMM colonies derived from bone marrow or peripheral blood, which have a very low recloning frequency. We showed that CFU-GEMM colonies from HUCB grown in the presence of SCF did not contain significant numbers of cells that could reclone. In contrast, however, Carrow et al. (1991) demonstrated a higher recloning frequency when the primary colonies were derived from cultures stimulated with erythropoietin and rMuSCF.

There are a number of explanations for the differences between our results and those in this study. The culture conditions we used differed in that G-CSF and GM-CSF were included in our experiments, together with erythropoietin and SCF. It is possible that the balance between self-renewal and terminal differentiation was different under our conditions and led to rapid depletion of CFC. In our experiments, CFU-GEMM colonies grown in the presence of SCF contained 100-fold more cells than those grown in the absence of SCF (Cicuttini et al., 1992a). It may be that these large CFU-GEMM colonies were due to limited self-renewal during the early phase of colony formation. The progeny CFC of these early divisions may have been recruited into the formation of a large "aggregate" colony. This possibility would be compatible with the observations of Carrow et al., (1991) and supports the notion that the CFU-GEMM have a higher proliferative potential than the majority of BM CFU-GEMM.

### In Vitro Stem-Cell Assays

**Secondary CFU (delta) assay for early hemopoietic cells** The secondary CFU assay involves the short-term (7-day) suspension culture of bone marrow depleted of committed progenitors and enriched for early stem cells in the presence of various cytokine combinations. The ability of these cytokines to promote survival, recruitment, differentiation, and expansion of stem cells and progenitor cells is measured in a secondary clonogenic assay. This secondary recloning assay has been used in a number of experimental settings to determine whether it can provide better information on the quality of cells in a transplant or chemotherapy regeneration situation (Juttner et al., 1985). We have used this assay to examine subpopulations of hemopoietic cells from HUCB.

Rhodamine-123 (Rh-123) is a supra-vital fluorochrome dye that is a useful tool for the hierarchical ordering of transplantable hematopoietic stem-cell, populations (Fauser and Messner, 1979). Recent studies have demonstrated that the most primitive cells in murine bone marrow have a low uptake of Rh-123, that is, Rh-123$^{low}$ (Bertoncello et al., 1985; Ploemacher and Brons, 1989). The potential of Rh-123 to distinguish between functionally distinct but closely related populations of primitive human hematopoietic cells has recently been examined. Double staining of human bone marrow with Rh-123 and phycoerythrin-labeled anti-CD34 Ab has demonstrated that the most primitive human hematopoietic cells are CD34+ Rh-123$^{low}$ (Udomsakdi et al., 1991).

We subdivided the CD34 HUCB cells on the basis of Rh-123 staining and examined single cytokines and cytokine combinations to investigate whether they could maintain survival, differentiation, and expansion of progenitor cells in the secondary CFC assay (Cicuttini, unpublished observation). Although an expansion of clonogenic cells was observed in cultures of the CD34+ Rh-123$^{high}$ and the total CD34+ cells, the CD34+ Rh-123$^{low}$ cells were found o produce the largest expansion of clonogenic cells, with Epo and SCF resulting in the most marked amplification. Following 7-day culture with combination of IL-3, IL-6, and SCF, the CD34+

Rh-123$^{low}$ cells resulted in a 50-fold expansion of CFC compared to an 0.8- and 0.9-fold expansion for the CD34+ Rh-123$^{high}$ and the total CD34+ cells. This is a larger expansion than was recently described for adult peripheral blood CD34+ cells cultured in a 6-factor combination for 7 days, where a 39-fold expansion was measured (Haylock et al., 1992). These results show that the CD34+ Rh-123$^{low}$ population contains the more immature progenitor cells with a large capacity for maintenance and expansion of clonogenic cells. These data also support the notion that fetal (cord blood) progenitor cells have a greater potential for expansion than adult progenitor cells.

**Long-term culture-initiating cells** *In vitro* hemopoiesis for weeks or months can be achieved in culture systems in which primitive hemopoietic stem cells are maintained in intimate relationship with a layer of marrow-derived stromal cells. These long-term bone marrow cultures (LTBMC) were first developed to support murine stem cells (CFU-S) replication (Dexter et al., 1977) and were subsequently adapted to sustain long-term hemopoiesis with marrow derived from other species including humans (Moore and Sheridan, 1971; Moore et al., 1979). Long-term culture is a more reliable measure of primitive progenitor cells than CFU-GM assay (Sutherland et al., 1989). Unlike normal bone marrow cells, cord blood cannot be established in primary long-term culture because it does not contain sufficient stromal precursor cells to provide the microenvironment necessary for self-renewal and differentiation of hemopoietic cells (Hows et al., 1992). However, when preformed marrow stroma was provided, CD34+ cells from HUCB could produce CFC for many weeks in culture (Cicuttini et al., 1992b). Indeed, the amplitude and length of progenitor cell production from cord blood in culture were shown to be superior to those of normal bone marrow, suggesting that the proportion of marrow "stem" cells is greater (Hows et al., 1992).

Long-term culture-initiating cells (LTC–IC) are defined, and can be assayed, by their ability to sustain prolonged (weeks) hemopoiesis and progenitor cell generation when inoculated onto preformed irradiated marrow stroma. In recent studies, the combination of negative selection by depletion of lineage positive cells, followed by positive selection for CD34+ cells exhibiting low right angle and low forward light-scatter properties, has permitted the enrichment of a population of cells possessing long-term culture-initiating properties (Andrews et al., 1989, 1990; Brandt et al., 1990; Verfaillie et al., 1990). We examined the various CD34+ subpopulations in human cord blood for the presence of LTC-IC by using stromal-cell lines (SCL) in which the rate of proliferation could be controlled by altering the zinc concentration (Cicuttini et al., 1992b). As we have reported (Cicuttini et al., 1992b), suppression of SCL proliferation by removal of zinc (Zn) made it possible to use these lines in co-culture with purified CD34+ subpopulations of cells. The line used, SCL 11, has been shown to produce GM-CSF, G-CSF, IL-6, and SCF/KL, but no IL-3 (Cicuttini et al., 1992b). CFC could be measured for over 2 months in co-cultures of SCL11 and CD34+ Rh-123$^{low}$ cells compared to only 40 days in cultures of CD34+ Rh-123$^{high}$. Significantly more total CFC were generated in co-cultures of the CD34+ Rh-123$^{low}$ cells. As was previously observed for human bone marrow (Udomsakdi et al., 1991) and adult peripheral blood (Udomsakdi et al., 1992), these results confirm that LTCIC are present within the CD34+ Rh-123$^{low}$ population from cord blood.

### B Lymphocytes

Human umbilical cord blood has been shown to be enriched for pre-B and B cells compared to adult peripheral blood. The mean frequency of pre-B cells has been shown to be 0.7% of total lymphocytes in cord blood compared to 0.2% in adult blood (Okino, 1987). The mean relative frequency of B lymphocytes in cord blood is also higher, being 11.4% of total lymphocytes compared to 5.4% in adult blood (Okino, 1987). In terms of absolute numbers of pre-B cells, cord blood contains ten times the number in adult blood.

The system of humoral immunity (B cells) develops early in gestation (Pabst and Kreth, 1980), but is not fully developed until after birth. Cord blood B lymphocytes display unique phenotypic and functional characteristics. The antigens CD1C, CD38, CD5, and CD23 are highly expressed on cord blood B cells but are expressed on only a small percentage of circulating B cells in normal adults (Small et al., 1989). Approximately half of the surface Ig$^{+}$ B lymphocytes express the CD5 Ag (Antin et al., 1986; Bofil et al., 1985; Durandy et al., 1990). The significance of the presence of CD5 on newborn B lymphocytes remains unclear. CD5+ B lymphocytes, which were first described in fetal spleen

(Antin et al., 1986) and cord blood (Bofil et al., 1985), are not found at such high levels in adulthood. However, CD5+ cells are increased in pathological conditions such as autoimmune disease or lymphoproliferative diseases (Boumsell et al., 1980; Plater-Zyberg et al., 1985; Hardy and Hayakawa, 1986; Sthoeger et al., 1989).

In addition to CD5 expression, a significant proportion of both CD5+ and CD5− newborn B cells express activation antigens (Durandy et al., 1990). Half of the sIg+ lymphocytes express the transferrin receptor and 10% the antigen defined by the Bac-1 mAb, both being early activation markers; (Larrick and Cresswell, 1980; Haynes et al., 1981). CD23, which has been shown to be a low-affinity Fc receptor of IgE (Bonnefoy et al., 1987), is also found on a significant number of B cells (Durandy et al., 1990). The detection of CD23 on normal resting cord blood B cells may not always reflect B-cell activation (Small et al., 1989). In this respect, it resembles the discontinuous expression of CD38 during normal B-cell ontogeny. CD38 is not only seen on resting and circulating cord blood B cells, but also on terminally differentiated plasma cells, with minimal expression on resting, circulating adult B cells (van Camp et al., 1982; Tedder et al., 1984; Small et al., 1989).

Despite the increased numbers of B cells in cord blood, Ig secretion is generally lower than in the adult. Cord blood B cells proliferate normally to *Staphylococcus aureus* Cowan strain 1 (SAC) and secrete IgM in response to a variety of polyclonal B-cell activators. However, very low immunoglobulin secretion is seen is pokeweed-mitogen- (PWM) stimulated cord blood mononuclear cells (Wu et al., 1976; Hayward and Lawton, 1977). Minimal or no IgG or IgA is produced following stimulation with pokeweed mitogen (Small et al., 1990) and SAC, and normal adult B cells, in response to these mitogens, produce both isotypes. Low immunoglobulin secretion (IgM with few IgG and almost no IgA) is found in B-cell assays that, unlike the PWM system, do not depend on a cell contact-mediated helper T-cell activity. These studies describe activation of isolated B cells with SAC or anti-μ antibodies plus various cytokines (Durandy et al., 1990). In contrast to the low levels of IgA and IgG secretion, cord blood B cells do switch to IgE-producing cells in response to interleukin-4 (IL-4) *in vitro* (Pastorelli et al., 1990). The minimal levels of IgE production measured in cord blood (less than 1 U/ml) are not due to a switching defect in cord blood B cells, but may be associated with the failure of cord blood T cells to produce detectable levels of IL-4.

Until recently, no *in vitro* B cell system was able to produce adult levels of IgG and IgA responses in newborn B cells. Recent studies suggest that whereas cord blood B cells are probably functionally naive, their inherent potential to switch to downstream isotypes approaches that of adult B cells. Tucci et al. (1991) examined isotype secretion in a culture system in which a direct cell contact with mutant EL4 thymoma cells, in conjunction with human T-cell supernatants, could lead to B-cell activation. It was shown that cord B cells, if provided with the extremely potent signals supplied by the mutant EL4 thymoma and T-cell supernatants, did reveal a potential for differentiation that was both qualitative and quantitatively indistinguishable from adult B cells. Hirohata et al. (1988) described a method for generating high-rate immunoglobin production from human B cells by co-culturing them with T cells that were stimulated with immobilized antibody to CD3. The production of both IgG and IgA, in addition to IgM, was observed, although the amounts of the switched isotypes produced were small by comparison to adult B cells. More recently, the frequency of B cells responding in this system was estimated and the factors modifying the response determined (Amoroso and Lipsky, 1990). The optimal response was observed with the addition of IL-2 and accessory cells when B-cell responses to anti-CD3-activated normal T cells reached a precursor frequency of 60–80%. Immunoglobin secretion of multiple isotypes was observed in wells where single B cells were seeded, indicating that class switching was occurring in these cultures. This contact-dependent activity expressed by activated TH cells has been shown to be due to triggering of the CD40 molecule via its ligand, CD40L, a 33-kD membrane protein (Brian, 1988; Armitage et al., 1992).

In summary, cord blood is enriched for pre-B and B lymphocytes compared to adult peripheral blood. Neonatal B lymphocytes display unique phenotypic and functional characteristics. The antigens CD1C, CD38, CD5, and CD23 are highly expressed on cord blood B cells but are normally expressed on only a small percentage of circulating B cells in normal adults. Recent studies suggest that whereas neonatal B cells are probably functionally naive, their inherent potential for stimulation approaches that of adult B cells. This can be realized as long as sufficiently strong T-cell help is available.

### T cells

**Phenotypic markers of differentiation** The percentage of lymphocytes expressing the CD2, CD3, and CD8 markers are lower in cord blood than adult blood. However, due to the increased white cell count in cord blood, the absolute numbers of CD2+ and CD8+ cells are comparable (Gerli et al., 1989). In contrast, the percentages of CD4+ cells (helper/ inducer T cells) in cord blood and adult peripheral blood are similar, although the absolute numbers of CD4+ cells are higher in cord blood. Nevertheless, cord blood CD4+ cells are deficient in their ability to provide help for antibody production. The functional abnormalities of cord blood T cells can be summarized as a relative absence of helper activity (Anderson et al., 1983). The cellular basis for this functional deficit has been examined by analyzing the phenotypic properties and immunoregulatory functions of subsets of cord blood CD4 + cells.

Recently, surface markers have been identified that seem to distinguish between "naive" versus "memory" T cells (Kingsley et al., 1988; Notarangelo et al., 1988; Landesberg et al., 1988; Sanders et al., 1988; Bradley et al., 1989; Clement et al., 1990). In contrast to CD4+ cells from adults, greater than 90% of cord blood CD4 + cells express high levels of CD45RA and L-selectin (Leu-8) (Clement et al., 1990) and have low levels of CD45RO (Sanders et al., 1988). The CD45RA antigen is first expressed by T-lineage cells relatively late during their intrathymic maturation and continues to be expressed by most T cells in the immunologically naive neonate (Clement, 1992). However, with increasing age and antigenic exposure CD45RA- /RO+ cells become more prevalent in the circulation and comprise the majority of cells in tissues. Analysis of the functional capabilities of CD4+CD45RA+ and CD4CD45RO+ cells has shown that proliferative responses to "memory" recall antigens, or the ability to provide help for antibody production, correlate with CD4+CD45RA-/RO+ cells (Clement et al., 1990). The major immunoregulatory functions described for CD4+CD45RA+ cells involve inhibition of immune responses, either directly or via the induction of suppressive activity by CD8+ cells.

Although cord blood CD4+CD45RA+ and CD4+CD45Ra- cells share certain properties with the analogous subsets in adults, the dominant immunoregulatory phenotype of cord blood CD4+ cells has been shown to be largely immunosuppressive, this phenotype being essentially consistent with the preponderance of CD4+CD45RA+ (and CD38+) cells (Tosato et al., 1980; Jacoby and Oldstone, 1983; Clement et al., 1990). Cord blood CD4 + cells cultured with adult B cells and PWM, or anti-CD4 + mAb, demonstrated no helper function (Clement et al., 1990). However, after activation with PHA and culture in IL-2, cord blood CD4+ + CD45RA+ cells acquired the ability to provide help for B-cell differentiation. This functional maturation was accompanied by conversion to the CD4+CD45RA-CD45RO+ phenotype.

However, when the small number of CD4+CD45RA-CD45RO+ cells in cord blood were purified and similarly analyzed, helper activity comparable to that of adult CD4+CD45RA- cells was found (Clement et al., 1990). This helper function was blocked by the presence of even small numbers of cord blood (but not adult) CD4+CD45RA+ cells. Irradiation or mitomycin C treatment of cord blood CD4+CD45RA+ cells abrogated their suppressive activity, but did not induce helper capability. In view of these phenotypic and functional differences between cord blood and adult CD4+CD45RA+ cells, it has been proposed that "naive" CD4+CD45RA+ cells undergo age-related maturational changes that are unrelated to their postulated activation-dependent post-thymic differentiation into CD4+CD45RA- "memory" cells capable of helper functions (Clement et al., 1990).

**Cytokine production** CD4 + T lymphocytes in the mouse were originally divided into at least two functionally distinct subsets base upon the pattern of lymphokines secreted (Mosmann and Coffman, 1989; Street and Mosmann et al., 1991). One subset referred to as TH1 cells produce IL-2, IFN-gamma and lymphotoxin upon activation. A second subset, type 2 helper T (TH2) cells, secrete IL-4, IL-5, IL-6, and IL-10. Both TH types express IL-3, GM-CSF, and TNF. The action of murine TH1 and TH2 T-cell subsets appeared to be mutually antagonistic. The TH1 and TH2 phenotypes were originally defined as very distinct cytokine secretion patterns expressed by large numbers of independently derived T-cell clones in a mutually exclusive fashion. Since that original definition, it has become clear that a number of other cytokine-secretion phenotypes are displayed by T-cell clones in tissue culture, and some of these phenotypes are stable and have been obtained in independent experiments (Kelso et al., 1991). A phenotype known as TH0 is characterized by the simultaneous secretion of IL-2, IL-4, IL-5,

IL-10, and IFN-gamma. It is thought that the TH0 phenotype represents a transient multipotential stage of CD4+ T cells that may differentiate further to the more restricted TH1 or TH2 phenotype. The THp precursor cell secretes only IL-2 on first contact with antigen. It is thought that the other phenotypes are derived either from this cell via a single step or, alternatively, that the exteme phenotypes, TH1 and TH2, are derived from intermediate phenotypes (Mosmann et al., 1991). Initially, it did not appear that human CD4+ cells could be separated into TH1 and TH2 cell types. However, there is now clear evidence for the differential production of lymphokines by human CD4+ T cells (Yessel et al., 1990).

The naive T lymphocytes found in cord blood produce a pattern of lymphokine production resembling that of THp cells upon restimulation, as, for example, by anti-CD3 antibodies *in vitro* (Ehlers and Smith, 1991). They respond to activation by proliferation, yet cannot secrete effector lymphokines other than IL-2. Cord blood T cells have a significantly enhanced proliferative responsiveness to IL-2 and an enhanced production of IL-2 compared to adult T cells (Rizzo et al., 1987; Fairfax and Borzy, 1988). In contrast, production of IL-4 by cord blood $CD4^+$ T cells, and IFN-gamma by cord blood CD4+ and CD8+ T cells, is less compared with analogous adult cell populations (Pastorelli et al., 1990; Lewis et al., 1991). Transcription of IL-4 is undetectable in cord blood T cells (TH2), and IFN-gamma transcription and IFN-gamma mRNA-containing cells (TH1) are reduced in cord blood T cells compared with adult T cells. Reduced lymphokine production by cord blood T cells correlates with their lack of a CD45RO (putative memory T-cell) population. Levels of IFN-gamma significantly higher than those produced by adult T cells, however, are synthesized in response to combinations of PHA and TPA, indicating that IFN-gamma production by cord blood T cells is not intrinsically defective. It might be speculated that the lack of B-cell help in cord blood T cells is a function of a more THp-like pattern of cytokine secretion.

In summary, the functional abnormalities of cord blood T cells can be summarized as dominance of suppression over helper activity with more than 90% of CD4+ T cells in cord blood expressing a naive phenotype, which may explain their helper activity. Their cytokine profiles suggest that they are THp cells.

**Natural Killer (NK) Cells**

Human NK cell activity develops gradually during intrauterine life (Uksika et al., 1983). Although cells bearing the NK marker CD57+ are negligible in cord blood (Abo et al., 1982), the proportions of CD16+ lymphocytes are equal to those of adult peripheral blood (Tarakkanan and Saksela, 1982; Perussia et al., 1983). The NK activity, of newborn peripheral blood is lower than that of adult blood (Uksila et al., 1982; Uksila et al., 1983) but can be enhanced by IL-2 (Ueno et al., 1985) and interferon (IFN) (Herberman and Ortaldo, 1981; Trinchieri and Perussia, 1984; Hercend and Schmidt, 1988). Although spontaneous NK activity of cord blood cells is profoundly reduced compared to the adult, antibody-dependent cellular cytotoxicity and NK-like activity generated in mixed lymphocyte cultures are similar in the two groups, with lymphokine-activated killer-cell (LAK) activity being higher in the neonate.

rHuIL-2 potentiates the cytotoxicity of cord blood CD16+ cell to approximately adult levels (Ueno et al., 1985) and significantly potentiates cytotoxic activity of both CD16 – cells from cord blood and adult blood that have no basal NK activity. IL-2 can also enhance depressed NK activity even at an early stage of human development, whereas NK boosting by IFN-gamma occurs effectively only during the latter half of gestation (Perussia et al., 1983). These results suggest that cord blood NK cells are heterogeneous and rIL-2 and gamma-IFN might potentiate the cytotoxicity of functionally immature NK cells or NK precursor cells. The existence of IL-2-sensitive NK precursors or functionally immature NK cells has been postulated, but the nature of these cells has not been determined (Uksila et al., 1982)

We have described the isolation of a unique subpopulation of CD7+ cells from human umbilical cord blood that in part corroborates and extends these earlier observations (Cicuttini et al., 1993). By immunorosette depleting of umbilical cord blood using T-cell, B-cell, granulocyte, macrophage, and hemopoietic progenitor cell (CD34) markers to isolate a lineage marker negative (Lin – ) population, a relatively homogeneous population was isolated with over 90% of cells expressing CD7. This Lin– CD7+ population was shown to be negative for all other T-cell markers tested (i.e., CD7 + 1 – 2 – 3 – 4 – 8 – ). However, approximately 30% of these cells were positive for the natural-killer (NK) cell-surface markers CD16 and CD56

(CD7 + NK + ). Both CD7 + NK + and CD7 + NK − populations proliferated in response to stimulation *in vitro* with IL-2/phytohemagglutinin (PHA)/PHA-conditioned medium. After such treatment, approximately 40% of the CD7+NK− acquired CD16 and CD56, and about 20% of the CD7+NK+ population became CD2+. The significance of the 60% of CD7+NK− cells that did not acquire other markers remains to be determined. In addition, although neither population was cytotoxic when first isolated, both populations acquired the ability to lyse the NK target cell line K562 when cultured under these conditions.

These data suggest that these two populations may represent a developmental sequence amongst NK cell precursors in human umbilical cord blood. We have clearly isolated precursor cells that antecede the acquisition of cytotoxic function. In particular, the CD7+NK− cells may be good candidates for the most immature NK precursor cells in cord blood and may represent cells recently released from bone marrow. Further analysis of such precursors may be useful in understanding the ontogeny of NK cells *in vivo*.

### Clinical Uses for Cord Blood

Bone marrow transplantation is now recognized as an effective form of treatment for an increasing number of malignant and nonmalignant disorders. Unfortunately, suitable marrow is frequently not available, with either the patient's own marrow being contaminated with tumor cells or potential allogenic marrow donors being unsuitable on the basis of HLA disparity or prior alloimmunization. Two-thirds of patients with leukemia and other life-threatening bone marrow diseases lack HLA-identical sibling marrow donors, and must rely on finding histocompatible donors through volunteer's registries (Beatty et al., 1988). Thus, many patients die or deteriorate before suitable transplant donors can be found (Ruuta et al., 1990). In addition, the risk of life-threatening acute graft-versus-host disease after unrelated marrow transplantation is high compared with the risk of HLA-identical sibling transplantation (Beatty et al., 1991).

HUCB may be one potential source of hemopoietic stem cells. HUCB is rich in hemopoietic progenitor cells, as measured in standard clonogenic assays for burst-forming units and granulocyte-macrophage colony-forming cells (Broxmeyer et al., 1989). HUCB has resulted in successful engraftment in a number of patients (Broxmeyer et al., 1991). Cryopreserved HUCB may be an alternative to bone marrow as a source of hemopoietic "stem" cells for HLA-identical transplantation. It has been suggested that a type cord blood bank would not only supplement the pool of registered marrow donors, especially in underrepresented ethnic and social groups, but could also potentially shorten the time between the initiation of an unrelated donor search and time of transplantation (Hows et al., 1992). Cord blood may be considered for treatment of leukemia, lymphoma, marrow-failure syndrome, and inborn errors of metabolism in patients who might benefit from myeloablative therapy and the infusion of normal hematopoietic stem cells.

A further use for HUCB is in gene therapy for hereditary deficiencies. The correction of certain human genetic defects might be possible by introducing functional genes into repopulating stem cells (Karson et al., 1992). The hemopoietic system is a primary target for attempting somatic gene therapy. Several leading laboratories recently have been able to demonstrate that bone marrow cells can be successfully transduced with foreign genes, resulting in the functional expression of these genes in culture. Retroviral vectors containing marker genes and the sequence for human proteins have been used to transduce cultured lymphocytes, which have then been reinfused into patients. Circulating hempoietic progenitor cells from human cord blood obtained at the time of term and premature deliveries as early as 19 weeks of gestation have been shown to express such transduced genes *in vitro*. Cord blood cells from fetal sheep sampled and transduced *in vitro* and transfused back *in utero* have been shown to express marker genes up to 2 years after birth. Although the efficiency of gene transfer into cells and their long-term expression need to be improved, the potential exists for treating some genetic diseases after prenetal diagnosis either *in utero* or shortly after birth (Karson et al., 1992)

*(Received October 10, 1993)*

*(Accepted December 3, 1993)*

### REFERENCES

Abo T., Cooper M.D., and Balch C.M. (1982). Post natal expansion of the natural killer and killer cell population in humans identified by the monoclonal HNK-1 antibody. J. Exp. Med. **155:** 321–326.

Allen T.D., and Dexter T.M. (1984). The essential cells of the hemopoietic microenvironment. Exp. Hematol. **12:** 517–521.

Amoroso K., and Lipsky P.E. (1990). Frequency of human B cells that differentiate in response to anti-CD3-activated T cells. J. Immunol, **145:** 3155–3161.

Anderson D.M., Lyman S.D., Baird A., Wignall J.M., Eisenman J., Rauch C., March C.J., Boswell H.S., Gimpel S.D., Cosman D., and Williams D.E. (1990). Molecular cloning of mast cell growth factor, a hematopoietin that is active in both membrane and soluble forms. Cell **63:** 235–243.

Andersson U., Britton S., De Ley M., and Bird G. (1983). Evidence for the ontogenic precedence of suppressor T cell functions in the human neonate. Eur. J. Immunol. **13:** 6–13.

Andrews R.G., Singer J.W., and Bernstein I.D. (1989). Precursors of colony-forming cells in humans can be distinguished from colony-forming cells by expression of the CD33 and CD34 antigens and light scatter properties. J. Exp. Med. **169:** 1721–1731.

Andrews R.G., Singer J.W., and Bernstein I.D. (1990). Human hematopoietic precursors in long-term culture: Single CD34+ cells that lack detectable T cell, B cell, and myeloid cell antigens produce multiple colony-forming cells when cultured with marrow stromal cells. J. Exp. Med. **172:** 355–358.

Antin J.H., Emerson S.G., Martin P., Gadol, N., and Ault K.A. (1986). Leu 1+ (CD5+) B cells: A major lymphoid subpopulation in human fetal spleen: Phenotype and functional studies. J. Immunol. **136:** 505–510.

Armitage R.J., Fanslow W.C., and Stockbine L. (1992). Molecular and biological characterization of a murine ligand for CD40. Nature **357:** 80–85.

Beatty P.G., Dahlber S., Michelson E., Mickelson E.M., Nisperos B., Opelz G., Martin P.J., and Hansen J.A. (1988). Probability of finding HLA matched unrelated donors. Transplantation **45:** 714–718.

Beatty P.G., Hansen J.A., Longton G.M., Thomas E.D., Sanders J.E., Martin P.J., Bearman S.I., Anasetti C., Petersdorf E.W., Mickelson E.M., Pepe M.S., Appelbaum F.R., Buckner C.D., Clift R.A., Peterson F.B., Stewart P.S., Storb R.F., Sullivan K.M., Tesler M.C., and Witherspoon R.P. (1991). Marrow transplantation from HLA matched unrelated donors for treatment of hematologic malignancies. Transplantation **51:** 443–447.

Berenson R.J., Andrews R.G., Bensinger W.I., Kalamasz D., Knitter G., Buckner C.D., and Bernstein I.D. (1988a). Antigen CD34+ marrow engraft lethally irradiated baboons. J. Clin. Invest. **81:** 951–955.

Berenson R.J., Andrews R.G., Bensinger W.I., Kalamasz D., Knitter G., Buckner C.D., and Bernstein I.D. (1988b). Autologous marrow transplantation in baboons and man using CD34+ stem cells. Exp. Hematol. **16** (Suppl.): 522.

Bertoncello I., Hodgson G.S., and Bradley T.R. (1985). Multiparameter analysis of transplantable hemopoietic cells. I. The separation and enrichment of stem cells homing to marrow and spleen on the basis of rhodamine-123 fluorescence. Exp. Hematol. **13:** 999–1006.

Bloom W., and Bartelmez G.W. (1940). Hematopoiesis in young human embryos. Am. J. Anat. **67:** 21–25.

Bofill M., Janossy G., Janossa M., Burford G.D., Seymour G.J., Wernet P., and Kelemen E. (1985). Human B cell development. II. Subpopulations in the human fetus. J. Immunol. **134:** 1531–1538.

Bonnefoy J.Y., Aubry J.P., Peronne C., Widjenes J., and Banchereau J. (1987). Production and characterization of a monoclonal antibody specific for the human lymphocyte low affinity receptor for IgE: CD23 is a low affinity receptor for IgE. J. Immunol. **138:** 2970–2978.

Boumsell L., Coppin H., Pham D., Raynal B., Lemere J. Dausset J., and Bernard A. (1980). An antigen-shared by a human T cell subset and B cell chronic lymphocytic leukemic cells. J. Exp. Med. **152:** 229–234.

Bradley L.M., Bradley J.S., Ching D.L., and Shiigi S.M. (1989). Predominance of T cells that express CD45R in the CD4+ helper/inducer lymphocyte subset of neonates. Clin. Immunol. Immunopathol. **51:** 426–435.

Brandt J., Srour E.F., Besien K.V., Briddell A., and Hoffman R. (1990). Cytokine-dependent long-term culture of highly enriched precursors of hematopoietic progenitor cells from human bone marrow. J. Clin. Invest. **86:** 932–941.

Brian A.A. (1988). Stimulation of B cell proliferation by a membrane-associated molecule from activated T cells. Proc. Natl. Acad. Sci. USA **85:** 564–568.

Broxmeyer H.E., Douglas G.W., Mangoe G., Cooper S., Bard J., English D., Asny M., Thomas L., and Boyse E.A. (1989). Human umbilical cord blood as a potential source of transplantable hemopoietic stem/progenitor cells. Proc. Natl. Acad. Sci. USA **86:** 3828–3837.

Broxmeyer H.E., Cooper S., Lu L., Hangoc G., Anderson D., Cosman D., Lyman S.D., and Williams D.E. (1991). Effect of murine mast cell growth factor (c-kit proto-oncogene ligand) on colony formation by human marrow hematopoietic progenitor cell. Blood **77:** 2142–2149.

Caracciola D., Clark S., and Rovera G. (1989). Differential activity of recombinant colony-stimulating factors in supporting proliferation of human peripheral blood and bone marrow myeloid progenitors in culture. Brit. J. Haematol. **72:** 306–311.

Carrow C.E., Hangoc G., Cooper S.H., Williams D.E., and Broxmeyer H.E. (1991). Mast cell growth factor (c-kit ligand) supports the growth of human multi-potential progenitor cells with a high replating potential. Blood **78:** 2216–2222.

Cicuttini F.M., Begley C.G., and Boyd A.W. (1992a) The effect of recombinant stem cell factor (SCF) on purified CD34-positive human hemopoietic progenitor cells derived from human umbilical cord blood. Growth Factors **6:** 31–39.

Cicuttini F.M., Martin M., Salvaris E., Ashman L., Begley G., Novotny J., Maher D., and Boyd A.W. (1992b). Support of early human cord blood progenitor cells on stromal cell lines transformed by SV40 large T antigen under the influence of an inducible (metallothionein) promoter. Blood **80:** 102–112.

Cicuttini F.M., Martin M., Petrie H., and Boyd A.W. (1993). A novel population of NK progenitor cells isolated from human umbilical cord blood. J. Immunol. **151:** 29–37.

Civin C.I., Banqerigo M.L., Strauss L.C., and Loken M.R. (1987). Antigenic analysis of hematopoiesis. VI. Flow cytometric characterization of My-10-positive progenitor cells in normal human bone marrow. Exp. Hematol. **15:** 10–17.

Clement L.T. (1992). Isoforms of the CD45 common leukocyte antigen family: Markers for human T-cell differentiation. J. Clin. Immunol. **12:** 1–10.

Clement L.T., Vink P.E., and Bradley G.E. (1990). Novel immunoregulatory functions of phenotypically distinct subpopulations of CD4+ cells in the human neonate. J. Immunol. **145:** 102–108.

Dexter T.M., Allen T.D., and Lajtha L.G. (1977). Conditions controlling the proliferation of hemopoietic stem cells in vitro. J. Cell. Physiol. **91:** 335–344.

Douay L., Gorin N.C., Mary J.-Y., Lemarie E., Lopez M., Najman A., Stachowaiak J., Giarratana M.C., Baillou C., and Salmon C. (1986). Recovery of CFU-GM from cryopreserved marrow and in vivo evaluation after autologous bone marrow transplantation. Exp. Hematol. **14:** 141–147.

Durandy A., Thuillier L., Forveille M., and Fischer A. (1990). Phenotypic and functional characteristics of human newborn B cells. J. Immunol. **144:** 60–65.

Ehlers S., and Smith K.A. (1991). Differentiation of T cell lymphokine gene expression: The in vitro acquisition of T cell memory. J. Exp. Med. **173:** 25–36.

Fairfax C.A., and Borzy M.S. (1988). Interleukin 2 production, proliferative response, and receptor expression by cord blood mononuclear cells. J. Clin. Lab. Immunol. **27:** 63–67.

Fauser A.A., and Messner H.A. (1979). Proliferative state of human pluripotent hemopoietic progenitors (CFU-GEMM) in normal individuals and under regenerative conditions after bone marrow transplantation. Blood **54:** 1197–1200.

Gerli R., Bertotto A., Crupi S., Arcangeli C., Marinelli I., Spinozzi F., Cernetti C., Angelella P., and Rambotti P. (1989). Activation of cord T lymphocytes. I. Evidence for a defective T cell mitogenes through the CD2 molecule. J. Immunol. **142:** 2583–2589.

Gupta S., Pahwa R., O'Reilly R., et al. (1976). Ontogency of lymphocyte subpopulation in human fetal liver. Proc. Natl. Acad. Sci. USA **73:** 919–922.

Hardy R.R., and Hayakawa K. (1986). Development and physiology of Ly1B and its human homolog, Leu1B. Immunol. Rev. **93:** 53–79.

Haylock D.N., To L.B., Juttner C.A., and Simmons P.J. (1992). Ex vivo expansion and maturation of peripheral blood CD34+ cells into the myeloid lineage. Blood **80:** 1405–1412.

Haynes B.F., Hemler M.E., Mann D.L., Fisenbarth G.S., Nichamer J., Mostowski H.S., Thomas C.A., Strominger J.L., and Fauci A.S. (1981). Characterization of a monoclonal antibody (4F2) that binds to human monocytes and to a subset of activated lymphocyte. J. Immunol. **126:** 1409–1414.

Hayward A.R., and Lawton A.R. (1977). Induction of plasma cell differentiation of human fetal lymphocytes: evidence for functional immaturity of T and B cells. J. Immunol. **119:** 1213–1217.

Herberman R.B., and Ortaldo J.R. (1981). Natural killer cells: Their role in defenses against disease. Science **214:** 24–30.

Hercend T., and Schmidt R.E. (1988). Characteristics and uses of natural killer cells. Immunol. Today **10:** 291–293.

Hirohata, S., Jelinek, D.F., and Lipsky, P.E. (1988). T cell dependent activation of B cell proliferation and differentiation by immobilized antibodies to CD3. J. Immunol. **240:** 3736–3744.

Hows J.M., Bradley B.A., Marsh J.C., Luft T., Continho L., Testa N.G., and Dexter T.M. (1992). Growth of human umbilical-cord blood in long-term haemopoietic cultures. Lancet **340:** 73–76.

Jacoby D.R., and Oldstone M.B.A. (1983) Delineation of suppressor and helper activity within the OKTA4-defined T lymphocyte subset in human newborns. J. Immunol. **131:** 1765–1770.

Juttner C.A., Haylock D.N., To L.-B., and Dyson P.G. (1990). Peripheral blood stem cell selection, collection and autotransplantation. Prog. Clin. Bio. Res. **333:** 447–459.

Karson E.M., Polvino W., and Anderson W.F. (1992). Prospects for human gene therapy. J. Reprod. Med. **37:** 508–514.

Kelso A., Troutt A.B., Maraskovsky E., Gough N.M., Morris L., Pech M.H., and Thomson J.A. (1991). Heterogeneity in Lymphokine Profiles of CD4+ and CD8+ T cells and Clones Activated in vivo and in vitro. Immunol. Rev. **123:** 85–114.

Kingsley G., Pitzalis C., Waugh A.P., and Panayi G.S. (1988). Correlation of immunoregulatory function with cell phenotype in cord blood lymphocytes. Clin. Exp. Immunol. **73:** 40–45.

Lajtha L.G. (1979). Stem cell concepts. Differentiation **14:** 23–33.

Landesberg R., Fallon M., and Insel R. (1988). Alterations in helper-inducer and suppressor-inducer T-cell subsets in human neonatal blood. Immunology **65:** 323–325.

Larrick J.W., and Cresswell P. (1980). Modulation of cell surface non transferrin receptors by cellular density and state of activation. J. Cell. Biochem. **11:** 579–586.

Leary A.G., Ogawa M., Strauss L.C., and Civin C.I. (1984). Single cell origin of multi-lineage colonies in culture. Evidence that differentiation of multipotent progenitors and restriction of proliferative potential of monopotent progenitors are stochastic processes. J. Clin. Invest. **74:** 2193–2197.

Lewis D.B., Yu C.C., Meyer J., English B.K., Kahn S.J., and Wilson C. (1991). Cellular and molecular mechanisms for reduced interleukin 4 and interferon-gamma production by neonatal T cells. J. Clin. Invest. **87:** 194–202.

Ma D.D., Varga D.E., and Biggs. J.C. (1987). Donor marrow progenitors (CFI-Mix, BFU-E and CFU-GM) and haemopoietic engraftment following HLA-matched sibling bone marrow transplantation. Leukaemia Res. **11:** 358–365.

McNiece I.K., Langley K.E., and Zsebo K.S. (1991). Recombinant human stem cell factor synergizes with GM-CSF, G-CSF, IL-3 and Epo to stimulate human progenitor cells of the myeloid and erythroid lineages. Exp. Hematol. **19:** 226–231.

Moore M.A.S., and Sheridan A.P. (1971). Conditions controlling the proliferation of hemopoietic stem cells in vitro. J. Cell. Physiol. **91:** 335–345.

Moore M.A.S., Sheridan A.P.C., Allen T.D., and Dexter T.M. (1979). Prolonged hematopoiesis in a primate bone marrow culture system: Characteristics of stem cell production and the hematopoietic microenvironment. Blood **54:** 775–795.

Mosmann T.R., and Coffman R.L. (1989). Heterogeneity of cytokine secretion patterns and functions of helper T cells. Adv. Immunol. **46:** 111–147.

Mosmann T.R., Schumacher, N.F., Budd R., O'Garra A., Fong T.A.T., Bond M.W., Moore K.W.K., Sher A., and Fiorentino D.F. (1991). Immunol. Rev. **123:** 209–229.

Diversity of cytokine synthesis and function of mouse CD4+ cells.

Nocka K., Buck J., Levi E., and Besmer P. (1990). Candidate ligand for c-kit transmembrane kinase receptor: KL, a fibroblast derived growth factor stimulates mast cells and erythroid progenitors. EMBO J. **9:** 3287–3294.

Notarangelo L.D., Panina P., Imberti L., Malfa P., Ugazio A.G., and Albertini A. (1988). Neonatal T4+ lymphocytes: Analysis of the expression of 4B4 and 2H4 antigens. Clin. Immunol. Immunopathol. **46:** 61–67.

Okino F. (1987). Pre-B cells and B lymphocytes in human cord blood and adult peripheral blood. Acta Paediatr. Jpn **29:** 195–201.

Pabst H.F., and Kreth H.W. (1980). Ontogeny of the immune response as a basis of childhood diseases. J. Pediatr. **97:** 519–534.

Pastorelli G., Rousset F., Pjene J., Peronne C., Roncarolo M.G., Tovo P. A., and der Vries J.E. (1990). Cord blood B cells are mature in their capacity to switch to IgE-producing cells in response to interleukin-4 in vitro. Clin. Exp. Immunol. **82:** 114–119.

Perussia B., Starr S., Abrahan S., Fanning V., and Trinchieri G. (1983). Human natural killer cells analyzed by B73.1, a monoclonal antibody blocking Fc receptor function. I. Characterization of the lymphocyte subset reactive with B73.1. J. Immunol. **130:** 2133–2141.

Plater-Zyberg C., Maini R.N., Lam K., Kennedy T.D., and Jannosy G. (1985). A rheumatoid arthritis B cell subset expressing a phenotype similar to that in chronic leukemia. Arthritis Rheum. **28:** 971–976.

Ploemacher R.E., and Brons R.H.C. (1989). Separation of CFU-S from primitive stem cells responsible for reconstitution of the bone marrow hemopoietic stem cell compartment following irradiation: Evidence for a pre-CFU-S cell. Exp. Hematol. **17:** 263–266.

Rizzo A.G., Ovak G.M., Donovan R.M., Chen P.B., Mookerjee B.K., and Pauly J.L. (1987). T Activation and long-term proliferation of human cord blood T cells with human recombinant interleukin-2. J. Clin. Lab. Immunol. **24:** 1–9.

Ruuta T., and Goldman J.M. (1990). Meeting report. A Nordic registry for unrelated volunteer donors? Bone Marrow Transplant **5:** 273–279.

Salahuddin S.Z., Markham P.D., Ruscetti F.W., and Gallo R.C. (1981). Long-term suspension cultures of human cord blood meyloid cells. Blood **58:** 931–938.

Sanders M.E., Makgoba M.W., Sharrow S.O., Stephany, D., Springer T.A., Young H.A., and Shaw S. (1988). Human memory T lymphocytes express increased levels of three cell adhesion molecules (LFA-3, CD2, and LFA-1) and three other molecules (UCHL1, CDw29, and Pgp-1) and have enhanced IFN-gamma production. J. Immunol. **140:** 1401–1407.

Small T.N., Knowles R.W., Keever C., Kernan N.A., Collins N., O'Reilly R., Dupont B., and Flomemberg N. (1987). M241 [CD1] expression on B lymphocyte. J. Immunol. **138:** 2864–2868.

Small T.N, Keever C., Collins N., Dupont B., O'Reilly R.J., and Flomenberg N. (1989). Characterization of B cells in severe combined immunodeficiency disease. Hum. Immunol. **25:** 181–193

Small T.N., Keever C.A., Weiner-Fedus S., Heller G., O'Reilly R.J., and Flomenberg N. (1990). B-cell differentiation following autologous, conventional, or T-cell depleted bone marrow transplantation: A recapitulation of normal-cell ontogeny. Blood **76:** 1647–1656.

Smith S., and Broxmeyer H.E. (1986). The influence of oxygen tension on the long-term growth in vitro of hemopoietic progenitor cells from human cord blood. Brit. J. Haematol. **63:** 29–34.

Sthoeger Z.M., Wakai M., Tse D.B., Vinciguerra V.P., Allan S.L., Budman D.R., Lichtman S.M., Schulman P., Weiselberg L.R., and Chiorazz I.N. (1989). Production of autoantibodies by CD5 expressing B lymphocyte from patients with chronic lymphocytic leukemia. J. Exp. Med. **169:** 255–268.

Street N., and Mossmann T.R. (1991). Functional diversity of T lymphocytes due to secretion of different cytokine patterns. FASEB J. **5:** 171–177.

Sutherland H.J., Eaves C.J., Eaves A.C., Dragowska W., and Landsdorp P.M. (1989). Characterization and partial purification of human bone marrow cells capable of initiating long-term hematopoiesis in vitro. Blood **74:** 1563–1570.

Tarakkanen J., and Saksela E. (1982). Umbilical-cord blood-derived suppressor cells of the human natural killer cell activity are inhibited by interferon. Scand. J. Immunol. **15:** 149–157.

Tedder T.F., Clement L.T., and Cooper M.D. (1984). Discontinuous expression of a membrane antigen (HB-7) during B lymphocyte differentiation. Tissue Antigens **24:** 140–149.

To L.B., and Juttner C.A. (1987). Peripheral blood stem cell autografting: A new therapeutic option for AML? Brit. J. Haematol. **66:** 285–288.

Tosato G.I., Magrath T., Koski I.R., Dooley J., and Blaese R.M. (1980). B cell differentiation and immunoregulatory T cell function in human cord blood lymphocytes. J. Clin. Invest. **66:** 383–388.

Trinchieri G., and Perussia B. (1984). Biology of disease: Human natural killer cells: biologic and pathologic aspects. Lab. Invest. **50:** 489–513.

Tucci A., Mouzaki A., James H., Bonnefoy J.Y., and Zubler R.H. (1991). Are cord blood B cells functionally mature? Clin. Exp. Immunol. **84:** 389–394.

Udomsakdi C., Eaves C.J., Sutherland H.K., and Lansdorp P.M. (1991). Separation of functionally distinct subpopulations of primitive human hematopoietic cells using rhodamine-123. Exp. Hematol. **19:** 338–342.

Udomsakdi C., Lansdorp P.M., Hogge D.E., Reid D.S., Eaves A.C., and Eaves C.J. (1992). Characterization of primitive hematopoietic cells in normal human peripheral blood. Blood **80:** 2513–2521.

Ueno Y., Miyawaki T., Seki H., A. Matsuda Taga K., Sato H., and Taniguchi N. (1985). Differential effects of recombinant human interferon-g and interleukin-2 on natural killer cell activity of peripheral blood in early human development. J. Immunol. **135:** 180–184.

Uksila J., Lassila O., and Hirvonen T. (1982). Natural killer cell function of human neonatal lymphocytes. Clin. Exp. Immunol. **48:** 649–654.

Uksila J., Lassila O., Hirvonen T., and Toivanen P. (1983). Development of natural killer cell function in the human fetus. J. Immunol. **130:** 153–156.

Van Camp B., Theilemans C., Dehou M.F., De Mey J., and De Waele M. (1982). Two monoclonal antibodies (OKTa1 and OKT10) for the study of the final B cell maturation. J. Clin. Immunol. **2:** 67s–74s.

Verfaillie C., Blakolmer K., and McGlave P. (1990). Purified primitive human hematopoietic progenitor cells with long-term in vitro repopulating capacity adhere selectively to irradiated bone marrow stroma. J. Exp. Med. **172:** 509–520.

Williams D.E., Lu L., and Broxmeyer H.E. (1987). Characterization of hematopoietic stem and progenitor cells. Immunol. Res. **6:** 294–304.

Wu L.Y.F., Blanco A., Cooper M.D., and Lawton A.R. (1976). Ontogeny of B-lymphocyte differentiation induced by pokeweed mitogen. Clin. Immunol. Immunopathol. **5:** 208–217.

Yessel M., Nakamoto T., Schneider P., Freitas V., Collins C., Webb D., Mensi N., Soderberg G., and Peltz G. (1990). Analysis of T lymphocyte clones from the synovial fluid and blood of a patient with lyme arthritis. Int. Immunol. **2:** 1081–1089.

